# Soluble PD-L1 is a predictive and prognostic biomarker in advanced cancer patients who receive immune checkpoint blockade treatment

**DOI:** 10.1038/s41598-021-99311-y

**Published:** 2021-10-05

**Authors:** So Yeon Oh, Soyeon Kim, Bhumsuk Keam, Tae Min Kim, Dong-Wan Kim, Dae Seog Heo

**Affiliations:** 1 Medical Oncology, Department of Internal Medicine, Pusan National University Yangsan Hospital, Yangsan, Republic of Korea; 2 Cancer Research Institute, Seoul National University and Integrated Major in Innovative Medical Science, Seoul National University College of Medicine, Seoul, Republic of Korea; 3 Biomedical Research Institute, Seoul National University, Seoul, Republic of Korea; 4 Department of Internal Medicine, Seoul National University Hospital, 101, Daehak-ro, Jongno-gu, Seoul, 03080 Republic of Korea

**Keywords:** Cancer, Oncology

## Abstract

Circulating soluble programmed death-1 ligand (sPD-L1) is measurable in the serum of cancer patients. This study aimed to investigate the significance of sPD-L1 in cancer patients receiving immune checkpoint inhibitor therapy. Blood samples were obtained before and after immune checkpoint inhibitor therapy (January 2015 to January 2019). The study cohort consisted of 128 patients who were diagnosed with non-small cell lung cancer (n = 50), melanoma (n = 31), small cell lung cancer (n = 14), urothelial carcinoma (n = 13), and other cancers (n = 20). Patients with a high level (> 11.0 pg/μL) of sPD-L1 were more likely to exhibit progressive disease compared with those with a low level (41.8% versus 20.7%, p = 0.013). High sPD-L1 was also associated with worse prognosis; the median PFS was 2.9 (95% confidence interval [CI] 2.1–3.7) months versus 6.3 (95% CI 3.0–9.6) months (p = 0.023), and the median OS was 7.4 (95% CI 6.3–8.5) months versus 13.3 (95% CI 9.2–17.4) months (p = 0.005). In the multivariate analyses, high sPD-L1 was an independent prognostic factor for both decreased PFS (HR 1.928, p = 0.038) and OS (HR 1.788, p = 0.004). sPD-L1 levels did not correlate with tissue PD-L1 expression. However, sPD-L1 levels were positively correlated with neutrophil to lymphocyte ratios and negatively correlated with both the proportion and the total number of lymphocytes. We found that high pretreatment sPD-L1 levels were associated with progressive disease and were an independent prognostic factor predicting lower PFS and OS in these patients.

## Introduction

Since the first immune checkpoint inhibitor (ICI), ipilimumab, was approved in 2011, the treatment paradigm for solid tumors has changed greatly. ICIs play important roles in the treatment of various types of solid tumors. However, in clinical practice, 16–55% of patients receiving ICI treatment suffer from severe toxicities^1^. Therefore, it is important to identify predictors of this negative response. Some biomarkers that predict treatment response have already been identified. For example, programmed death ligand 1 (PD-L1)^2^ expression in tumor tissue is predictive of a higher response rate to programmed death 1 (PD-1) or PD-L1 inhibitor therapy in patients with non-small cell lung cancer (NSCLC)^3–5^. It is also a marker of poor prognosis in some solid tumors^6–8^. However, inter-assay discordance and tumor heterogeneity hinder the standardization of PD-L1 testing and interpretation^9,10^. Researchers have tried to standardize the methods used to measure PD-L1 expression, but no clinically validated assays are currently available^11^. Microsatellite instability or deficient mismatch repair (dMMR) is one of the biomarkers studied as a predictor of ICI response^2^. dMMR is associated with favorable clinical outcomes in patients with colorectal cancer^12,13^. PD-1 blockade results in a robust response in some subjects with dMMR-positive solid tumors^14^. Tumor mutational burden, tumor infiltrating lymphocytes, and genetic signatures are also predictive factors. However, these markers lack standardization and exhibit high variability across tumor types and study settings^2^.

Lack of sufficient tissue for examination is another common and important factor limiting biomarker utility. A shortage of tumor tissue is especially problematic in patients with NSCLC because diagnostic biopsies often yield only tiny pieces of tissue. However, approximately five molecular genetic tests are required to select the appropriate therapeutic agents for an individual NSCLC patient^15,16^. The use of small biopsies can also result in misclassification of up to 35% of PD-L1 assessments in patients with advanced NSCLCs^17^. Therefore, circulating blood biomarkers are being investigated to predict the response to PD-1/PD-L1 blockade. These potential markers include, among others, circulating immune cells and circulating PD-L1^18–23^. In previous studies, CD8^+^ T cells showed a proliferative burst or functional reinvigoration after PD-1 blockade^24,25^. Another study showed that functionally active CD8^+^ T cells or NK cells are associated with good prognosis after PD-1 blockade^26^.

PD-L1 is present in a membrane-bound form in tumor cells and immune cells. However, it may also be secreted as a truncated form called soluble PD-L1 (sPD-L1) which may mediate immunosuppression or resistance to PD-L1 blockade therapy^27–29^. Compared with healthy subjects, circulating sPD-L1 concentrations are elevated in the plasma of patients with cancer. In patients with lymphoma, these concentrations return to normal levels after a complete response^30^. High concentrations of sPD-L1 are associated with poor prognosis in patients with hepatocellular carcinoma, gastric cancer, and NSCLC^31–34^. Here, we examined circulating sPD-L1 and its role as a prognostic and predictive marker in patients with cancer who received ICI treatment. We analyzed pretreatment and posttreatment levels of circulating sPD-L1 and investigated the relationship between these levels and clinical outcomes in patients with advanced solid tumors.

## Results

### Patient and sample characteristics

A total of 128 patients with stage IV solid tumors were included in this study. Samples were obtained between January 2015 and January 2019. The characteristics of the study population are presented in Table 1. The sample interval range was 14–49 days in 66 of the 67 patients with available pre- and posttreatment samples; in the remaining patient, the sample interval was 576 days (median, 21 days; range, 14–576 days; Supplementary Table 1). The objective response rate (ORR) was 18.8% (among 112 evaluable patients). The median progression-free survival (PFS) and overall survival (OS) were 4.2 months (95% CI 2.3–6.1 months) and 10.8 months (95% CI 7.9–13.8 months), respectively.

**Table 1:** Patient characteristics.

	N (%) or median(range)
Total	128 (100)
Age	62 (21–82) years
**Sex**	
Male	89 (69.5)
Female	39 (30.5)
**ECOG PS**	
0	27 (21.1)
1	98 (76.6)
2	1 (0.8)
Unknown	2 (1.6)
**Diagnosis**	
NSCLC	50 (39.1)
Melanoma	31 (24.2)
SCLC	14 (10.9)
UCC	13 (10.2)
RCC	6 (4.7)
HNSCC	5 (3.9)
Salivary gland cancer	4 (5.8)
Others	5 (3.9)
History of radiotherapy*	
Never irradiated	50 (39.1)
Received radiotherapy	78 (60.9)
Within 4 weeks to ICI treatment	14 (17.9)
Earlier than 4 weeks	43 (55.1)
After end of ICI treatment	21 (26.9)
**Type of ICI treated**^†^	
Monotherapy	
Nivolumab	41 (32.0)
Pembrolizumab	32 (25.0)
Durvalumab	15 (11.7)
Ipilimumab	5 (3.9)
Atezolizumab	4 (3.1)
Combination therapy	
Pembrolizumab/other	13 (10.2)
Atezolizumab/other	13 (10.2)
Nivolumab and ipilimumab	2 (1.6)
Others	3 (2.3)

*ECOG PS* eastern cooperative oncology group performance status, *NSCLC* non-small cell lung cancer, *SCLC* small cell lung cancer, *UCC* urothelial carcinoma, *RCC* renal cell carcinoma, *HNSCC* head and neck squamous cell carcinoma, *ICI* immune checkpoint inhibitor.

*Encompasses all types of radiotherapy, including stereotactic radiosurgery.

^†^Denotes patients who received ICI as part of a clinical trial.

### Pretreatment sPD-L1 level and response

The mean pretreatment sPD-L1 level was 13.5 ± 12.1 pg/μL and the median level was 11.0 pg/μL (range, 3.2–122.1 pg/μL). The mean sPD-L1 value in the patients with cancer was not significantly different from the mean level in healthy volunteers (13.5 pg/μL versus 10.6 pg/μL, respectively; p = 0.312, *t* test). However, the mean sPD-L1 concentration was numerically higher in cancer patients and we have to consider the possibility that there was no statistical significance because of the small number of healthy volunteers (n = 20). We used a receiver operating characteristic (ROC) curve to determine the optimal sPD-L1 cutoff level for the prediction of progressive disease after ICI treatment (Supplementary Fig. 2). We found that a cutoff value of 11.0 pg/μL distinguished best between patients who showed progressive disease after ICI treatment versus those who did not have progressive disease (sensitivity, 65.7%; specificity, 60.3%). The area under the curve value was 0.668 (95% confidence interval (CI) 0.568–0.769; p = 0.004). This cutoff value was used for subsequent statistical analyses. We used *χ*^2^ tests to compare treatment response in patients with varying sPD-L1 levels (low versus high). The ORRs were not significantly different (23.4% versus 22.7% in the low and high groups, respectively; p = 0.573). However, the disease control rates were 79% versus 58% in patients with low and high sPD-L1 levels, respectively (p = 0.013) (Table 2). The relationship between sPD-L1 levels and treatment response is illustrated with a bar chart in Supplementary Fig. 3.

**Table 2:** Response according to pretreatment sPD-L1 levels. Response was evaluated in 113 patients. The results are presented as total numbers (percentages).

	ICI response		Total	p value*
	CR, PR, SD	PD		
Low sPD-L1	46 (79)	12 (21)	58 (100)	0.013
High sPD-L1	32 (58)	23 (42)	55 (100)	
Total	78	35	113	

*sPD-L1* soluble programmed death ligand-1, *ICI* immune checkpoint inhibitor, *CR* complete response, *PR* partial response, *SD* stable disease, *PD* progressive disease.

*Significance at p < 0.05 (*χ*^2^ test).

### Pretreatment sPD-L1 levels and prognosis

**Figure 1:**
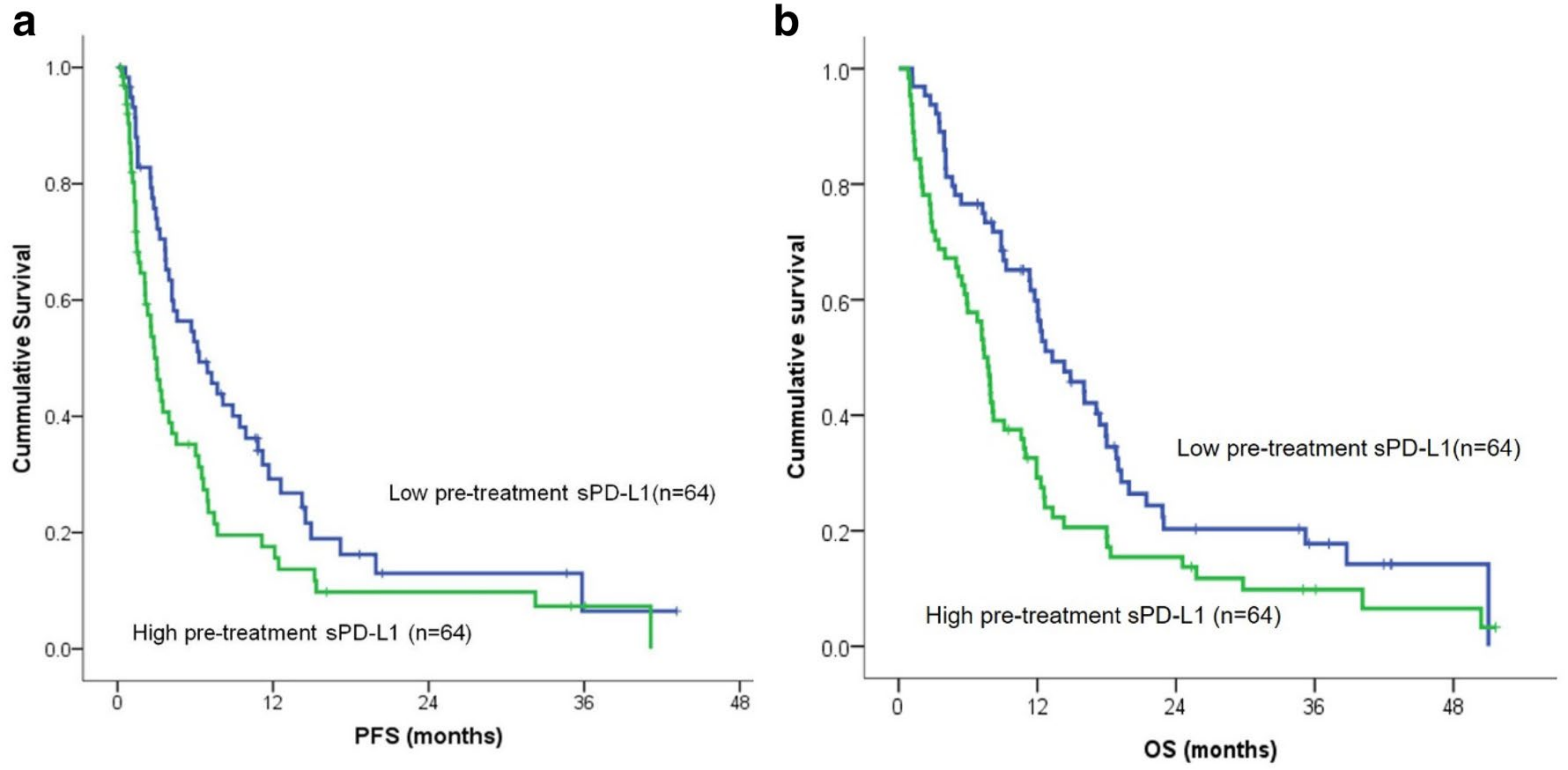
Kaplan–Meier curves for progression-free survival (**a**) and overall survival (**b**) stratified by soluble programmed death ligand 1 level. *PFS* progression-free survival, *OS* overall survival.

PFS varied between patients with low versus high levels of sPD-L1. The median PFS was 6.3 months (95% CI 3.0–9.6 months) versus 2.9 months (95% CI 2.1–3.7 months); this difference was statistically significant (p = 0.023, log-rank test; Fig. 1a). OS was also significantly different between the two groups. The median OS was 13.3 months (95% CI 9.2–17.4 months) versus 7.4 months (95% CI 6.3–8.5 months) (p = 0.005, log-rank test; Fig. 1b) in the low and high sPD-L1 groups, respectively. Univariate and multivariate analyses were performed to investigate potential correlations between sPD-L1 levels, clinical factors, and PFS and OS (Table 3). The results of the univariate analysis indicated that performance status, tissue PD-L1 expression, neutrophil to lymphocyte ratio (NLR), serum albumin levels, and sPD-L1 levels were significant predictors of PFS. Performance status, NLR, platelet count, serum albumin levels, total serum protein levels, and sPD-L1 levels were significant predictors of OS. The PFS and OS of patients who received radiation prior to ICI therapy were not significantly different from those who did not. Factors with p-values < 0.05 were included in the multivariate analysis. The multivariate analysis found that tissue PD-L1 expression and sPD-L1 levels were significant predictors of PFS. NLR, total serum protein levels, and sPD-L1 levels were significant predictors of OS (Table 3).

**Table 3:** Univariate and multivariate analysis of PFS and OS*

	N = 128	PFS				OS			
		Univariate analysis		Multivariate analysis		Univariate analysis		Multivariate analysis	
		Median (95% CI) (months)	p value	Exp(B) (95% CI)	p value	Median (95% CI) (months)	p value	Exp(B) (95% CI)	p value
**Age (years)**									
< 65	78	3.4 (2.4–4.4)	0.191			8.2 (5.4–11.0)	0.387		
≥ 65	50	7.3 (5.8–8.7)				14.9 (10.5–19.2)			
**Sex**									
M	89	3.7 (2.4–5.0)	0.163			9.1 (6.0–12.3)	0.122		
F	39	6.3 (2.4–10.2)				12.1 (9.1–15.1)			
**ECOG PS**									
0	28	7.7 (0.8–14.6)	0.012	1.223 (0.560–2.671)	0.614	12.7 (0–27.3)	0.036	1.402 (0.852–2.308)	0.184
1	100	4.0 (3.1–4.8)				10.6 (7.2–14.0)			
**Radiation therapy**									
Prior to ICI	57	3.7 (2.2–5.2)	0.056			8.1 (3.4–12.9)	0.058		
After ICI or never irradiated	71	6.2 (3.3–9.0)				12.3 (8.1–15.9)			
**Tissue PD-L1 IHC**									
Negative, weak	43	3.0 (2.4–3.7)	0.021	2.232 (1.119–4.453)	0.023	8.2 (3.4–13.0)	0.841		
Moderate/strong	25	6.9 (5.8–8.1)				12.6 (6.0–19.1)			
Unknown	60	–				–			
**NLR**									
< 2.8	62	5.7 (3.8–7.6)	0.042	1.055 (0.531–2.099)	0.878	14.3 (9.5–19.2)	< 0.001	1.913 (1.242–2.946)	0.003
≥ 2.8	66	3.3 (1.8–4.7)				7.2 (4.2–10.1)			
**Platelet count**									
< 250k	67	6.3 (3.4–9.2)	0.065			12.4 (10.4–14.4)	0.016	1.474 (0.990–2.195)	0.056
≥ 250k	61	3.3 (2.0–4.6)				7.7 (6.7–8.7)			
**Serum albumin (g/dL)**									
< 4.0	70	2.8 (2.1–3.5)	0.047	1.788 (0.908–3.521)	0.093	6.8 (4.3–9.3)	0.006	1.083 (0.690–1.700)	0.729
≥ 4.0	58	6.9 (5.5–8.4)				14.3 (9.9–18.8)			
**Serum total protein (g/dL)**									
< 7.2	65	3.4 (2.5–4.3)	0.078			8.0 (6.5–9.4)	0.009	1.766 (1.148–2.719)	0.010
≥ 7.2	63	6.2 (2.7–9.7)				12.7 (9.6–15.8)			
**Glucose (mg/dL)**									
< 126	85	3.7 (2.5–4.8)	0.124			11.0 (7.2–14.8)	0.808		
≥ 126	41	6.5 (3.3–9.7)				12.3 (8.1–16.5)			
Unknown	2	–				–			
**sPD-L1 level (pg/μL)**									
< 11	64	6.3 (3.0–9.6)	0.023	1.928 (1.038–3.581)	0.038	13.3 (9.2–17.4)	0.005	1.788 (1.207–2.650)	0.004
≥ 11	64	2.9 (2.1–3.7)				7.4 (6.3–8.5)			

*PFS* progression-free survival, *OS* overall survival, *CI* confidence interval, *ECOG PS* eastern cooperative oncology group performance status, *ICI* immune checkpoint inhibitor, *PD-L1* programmed death ligand-1, *IHC* immunohistochemical stain, *NLR* neutrophil to lymphocyte ratio, *sPD-L1* soluble programmed death ligand-1.

*Significant when p value is less than 0.05. Variables with *p* < 0.05 were examined in the multivariate analyses.

### Changes in sPD-L1 levels after treatment

**Figure 2:**
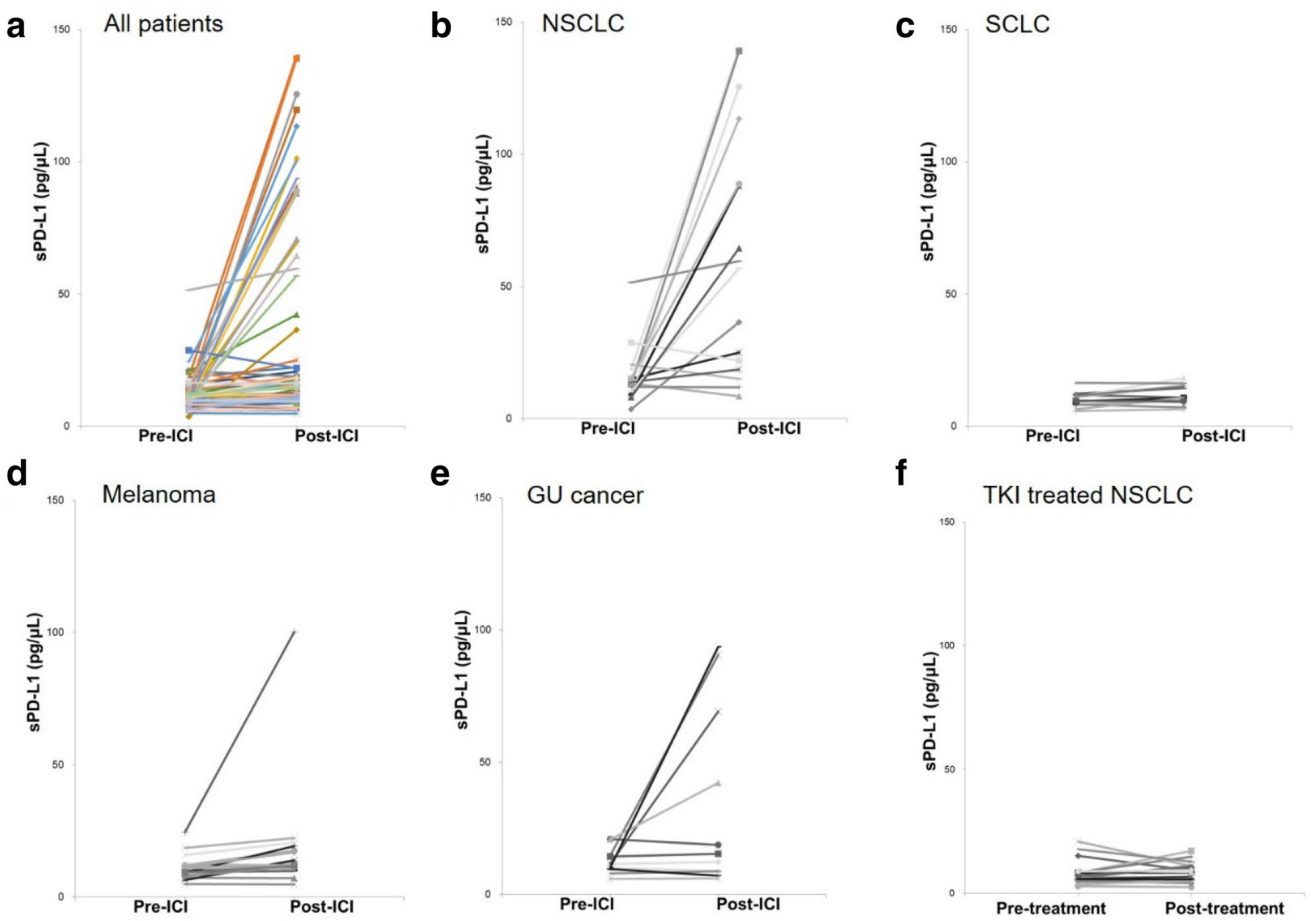
Changes in sPD-L1 levels before and after treatment, according to cancer type and treatment type in all patients (**a**), NSCLC patients (**b**), SCLC patients (**c**), melanoma patients (**d**), GU cancer patients (**e**), and NSCLC patients treated with TKIs (**f**). The median time points for post-ICI sampling were 15 days (NSCLC), 21 days (melanoma and GU cancer), and 42 days (SCLC). *Pre-ICI* before administration of immune checkpoint inhibitor, *Post-ICI* after administration of immune checkpoint inhibitor, *NSCLC* non-small cell lung cancer, *SCLC* small cell lung cancer, *GU* genitourinary, *TKI* tyrosine kinase inhibitor.

**Figure 3:**
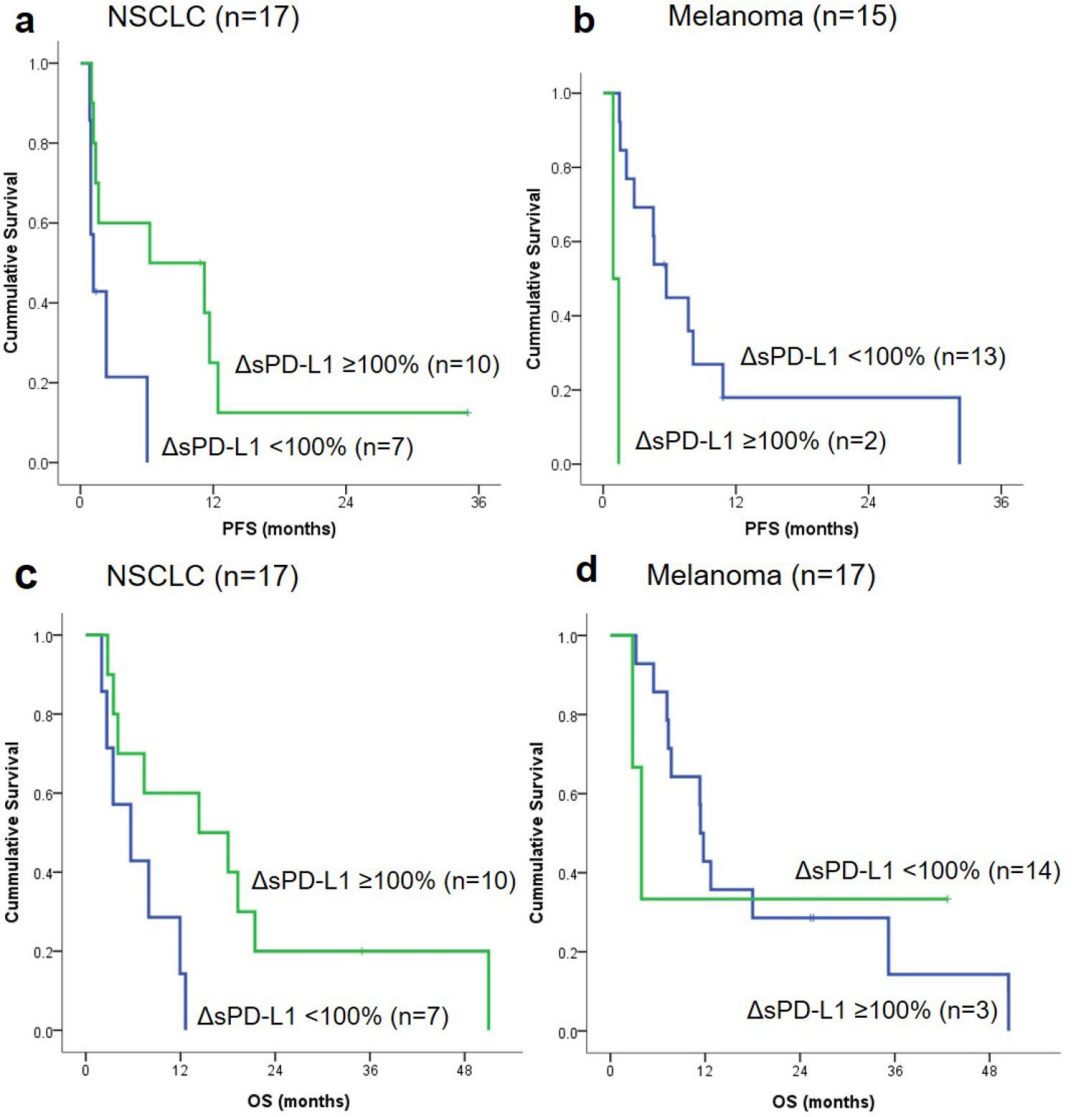
Kaplan–Meier curves for progression-free survival and overall survival in patients with NSCLC (**a**,**c**) and patients with melanoma (**b**,**d**) stratified by soluble programmed death ligand 1 levels. *NSCLC* non-small cell lung cancer, *ΔsPD-L1* change in sPD-L1 concentration between pretreatment and posttreatment sampling, *PFS* progression-free survival, *OS* overall survival.

We analyzed the changes in sPD-L1 concentrations in 67 patients with available pre- and posttreatment samples. sPD-L1 levels generally increased with ICI administration during the 2- to 7-week period following the start of treatment. However, the changes in sPD-L1 concentration (ΔsPD-L1) varied (Fig. 2a), and the patterns of change were somewhat different for each cancer type (Figs. 2b, 3e). A sharp increase was apparent in some patients with NSCLC or genitourinary cancer (Fig. 2b,e). However, with one exception, the amplitude of change was negligible in SCLC and melanoma patients (Fig. 2c,d). For comparison, we analyzed ΔsPD-L1 between pre- and posttreatment samples from NSCLC patients treated with tyrosine kinase inhibitors. The results indicated that the pattern of change in NSCLC patients treated with tyrosine kinase inhibitors was very similar to that of the SCLC patients (Fig. 2f). Interestingly, the pattern of change was quite different among the ‘immunogenic’ tumor types, such as melanoma, NSCLC, and genitourinary tumors. Possible explanations for these different patterns include different sources of sPD-L1 or carcinoma-specific biological differences. These patterns might also have been affected by differences in sampling intervals; the median sampling interval for NSCLC patients was significantly shorter than the intervals for melanoma or SCLC patients (Supplementary Table 1).

### Relationship between changes in sPD-L1 levels and response or prognosis

The analyses to examine relationships between ΔsPD-L1 and treatment response did not reveal any apparent patterns (Supplementary Fig. 4 and Supplementary Table 2). There was no significant difference in pan-cancer PFS according to ΔsPD-L1. However, NSCLC patients with a > 100% increase in sPD-L1 levels after ICI treatment had longer PFS than patients without this increase (Fig. 3a); the median PFS was 6.3 months (95% CI 0.0–19.4 months) versus 1.2 months (95% CI 0.6–1.8 months), respectively (p = 0.029, log-rank test). The opposite was observed in patients with melanoma. The median PFS was 0.9 months (95% CI not available) versus 5.7 months (95% CI 3.8–7.6 months) in patients with ΔsPD-L1 of 100% or more versus those with ΔsPD-L1 less than 100% after ICI treatment (p < 0.001, log-rank test; Fig. 3b). The OS response was in the same direction as PFS but was not statistically significant in patients with melanoma. In patients with NSCLC, the median OS after ICI treatment was 14.3 months (95% CI 0.0–30.8 months) in patients with ΔsPD-L1 ≥ 100% versus 5.7 months (95% CI 0.0–11.4 months) in those with ΔsPD-L1 < 100% (p = 0.022, log-rank test; Fig. 3c). The median OS was 3.9 months (95% CI 2.1–5.7 months) versus 11.4 months (95% CI 10.6–12.2 months) (p = 0.827, log-rank test) in patients with melanoma (Fig. 3d).

### Correlation between sPD-L1 levels and tissue PD-L1 expression or circulating immune cells

**Figure 4:**
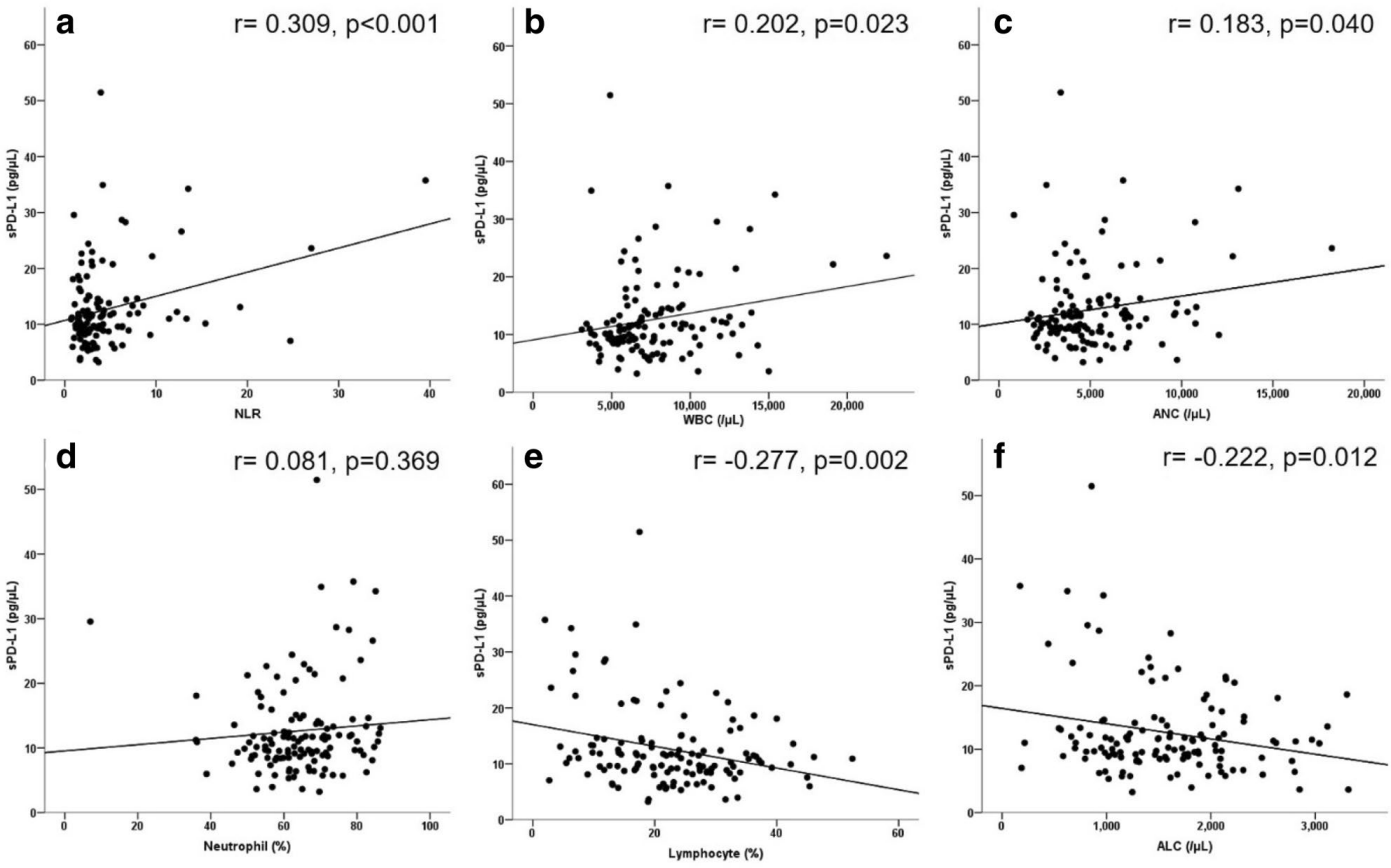
Correlation between sPD-L1 levels and circulating immune cells, including NLR (**a**), WBCs (**b**), ANCs (**c**), neutrophils (**d**), lymphocytes (**e**), and ALCs (**f**). Pearson’s correlation coefficients are denoted as r. *NLR* neutrophil to lymphocyte ratio, *WBC* white blood cell, *ANC* absolute neutrophil count, *ALC* absolute lymphocyte count.

We next examined the correlation between tissue PD-L1 expression and sPD-L1 levels. A Spearman’s rho value of 0.069 (p = 0.575) suggested that these two factors were not correlated. The mean sPD-L1 levels in the negative/low PD-L1 expression group were also not significantly different from those in the moderate/high expression group (12.0 ± 1.0 pg/μL versus 12.9 ± 1.2 pg/μL, respectively; p = 0.649, *t* test). We also examined the correlation between sPD-L1 levels and NLR or circulating immune cell numbers (Table 4 and Fig. 4). NLR, white blood cell count, and absolute neutrophil count (ANC) were positively correlated with sPD-L1 levels (Fig. 4a–c). The proportion and total number of lymphocytes were negatively correlated with sPD-L1 levels (Fig. 4e,f).

**Table 4:** Analysis of correlations between sPD-L1 and circulating immune cells. Two outliers with extremely high sPD-L1 levels or absolute neutrophil counts were excluded from these analyses.

	Pearson’s correlation coefficient	p value*
NLR	0.309	< 0.001
WBC	0.202	0.023
ANC	0.183	0.040
Neutrophil	0.081	0.369
Lymphocyte	− 0.277	0.002
ALC	− 0.222	0.012

*sPD-L1* soluble programmed death ligand-1, *NLR* neutrophil to lymphocyte ratio, *WBC* white blood cell count, *ANC* absolute neutrophil count, *ALC* absolute lymphocyte count.

*Significance at p < 0.05.

## Discussion

The results of this study suggest that pretreatment serum sPD-L1 concentrations can be used to predict treatment response, PFS, and OS in patients receiving ICI treatment for advanced solid tumors. We found that a high baseline sPD-L1 level predicted a low disease control rate. The pretreatment sPD-L1 level was an independent prognostic factor predicting PFS and OS, even after controlling for known prognostic variables. Many studies of sPD-L1 have been published in recent years. Most studies have found that high pretreatment sPD-L1 levels are associated with decreased survival in patients with advanced solid tumors (e.g., lung cancer, gastric cancer, renal cell carcinoma, melanoma, hepatocellular carcinoma, pancreatic cancer, and soft tissue sarcoma)^31,33,35–43^. High pretreatment sPD-L1 levels are also associated with a poor response to ICI treatment in patients with melanoma or lung cancer^36,44^. Therefore, it has been generally accepted that high pretreatment sPD-L1 levels are associated with a poor treatment response and a worse prognosis. The results of this study are consistent with those of previous studies in that pretreatment sPD-L1 levels had predictive and prognostic value for patients with advanced cancer.

We also investigated whether changes in sPD-L1 levels after ICI treatment are associated with response or prognosis. We were not able to acquire the sample sizes required to identify correlations between the extent of sPD-L1 changes and tumor response or PFS. Because each tumor type had very different patterns of change in sPD-L1 levels, the effects of changes in sPD-L1 on PFS were likely diluted. This possibility was supported by our observation of the opposite pattern when each cancer type (melanoma and NSCLC) was analyzed separately. Few studies have examined the relationships between sPD-L1 dynamics and prognosis in patients receiving radiation or chemoradiation, and the results of those limited studies have been inconsistent. Increased sPD-L1 levels after chemoradiation are associated with a poor prognosis in patients with rectal cancer^45^. In contrast, preliminary results indicate that in patients with biliary tract cancer, increased sPD-L1 levels after chemotherapy are associated with longer PFS^46^. In patients with advanced pancreatic cancer receiving cytotoxic chemotherapy, sPD-L1 dynamics correlate with the disease course^39^. Several studies have been performed to investigate the dynamics of sPD-L1 changes over time in patients receiving ICI treatment. One study of patients receiving ipilimumab-based treatment for advanced melanoma found that many who had a ≥ 1.5-fold increase in sPD-L1 within 4.5 months after treatment experienced progressive disease^44^. A study analyzed PD-L1 mRNA expression in plasma-derived exosomes in melanoma and NSCLC patients at baseline and 2 months after PD-1 inhibitor treatment. They showed that exosomal mRNA copies of PD-L1 were correlated with tumor response in melanoma (n = 18) and NSCLC (n = 8) patients^47^. Another study of 21 patients with lung, gastric, or bladder cancer who underwent anti-PD-1 therapy found that a reduction in plasma sPD-L1 levels is significantly correlated with tumor size reduction^48^. The significance of sPD-L1 in our NSCLC population was inconsistent with other studies. It seems too early to draw conclusions, as the sample size of all previously published studies was very small.

We also examined the association between PD-L1 expression levels in tumor tissue and circulating sPD-L1 levels. PD-L1 expression in tumor tissue did not correlate with sPD-L1 levels. This was an unexpected finding, as we expected the levels of sPD-L1 to reflect the expression of tissue PD-L1. Therefore, we explored circulating immune cells as a possible source of sPD-L1. sPD-L1 levels were positively correlated with NLR and negatively correlated with total lymphocyte numbers. The results of previous studies suggest that sPD-L1 originates mainly from a membrane-bound form of PD-L1 present in cancer cells or immune cells^37,43,49^. The results of our study suggest that sPD-L1 is derived from cells identified as neutrophils in routine complete blood count tests. Additionally, in a previous study that analyzed peripheral immune cells from 28 cancer patients, 5 to 35% of peripheral blood myeloid-derived suppressor cells (MDSCs) expressed PD-L1. The expression of PD-L1 is highest in granulocytic MDSCs (35.8%) and lower in T cells (< 1%), most NK cells (< 1%), and B cells (11%)^50^. Therefore, we can carefully assume that sPD-L1 originates from granulocytic MDSCs. Considering the limited information currently available, however, the main source of sPD-L1 should be explained in future studies.

This study had some limitations. The study population was heterogeneous with respect to cancer type, blood sampling times, ICI administration timing, and post-ICI treatment regimens. This heterogeneity might have reduced the power to detect the effects of various characteristics for individual types of cancer. There were also no reference levels for sPD-L1, and the results between assay kits seemed to be quite different. There are also no pre-established cutoff levels that predict response or prognosis. To overcome this problem, some researchers are investigating reproducible, standardizable methods that can be used instead of ELISA^49^.

In summary, high pretreatment sPD-L1 levels were associated with low disease control rates. sPD-L1 levels were an independent predictor of PFS and OS in patients receiving ICI treatment for advanced cancer. sPD-L1 was likely derived from peripheral blood neutrophils, and levels generally increased following ICI administration. The amplitude of sPD-L1 change after ICI treatment was associated with PFS in patients with NSCLC and melanoma but in the opposite direction for each cancer type. A future, larger study should be undertaken to reveal the significance of changes in sPD-L1 levels for each carcinoma type.

## Methods

### Patients

Blood samples were taken from each cancer patient before they received ICI treatment. Some patients participated in other, previously published studies^51–53^. Posttreatment samples were obtained from 67 patients at either the next visit or a later visit. Patients were eligible for the study if they (1) were 18 years of age or older, (2) had a histologically confirmed malignancy, (3) received ICI treatment at Seoul National University Hospital, (4) had study samples taken before and/or after ICI treatment, and (5) completed a written consent form for research using human derivatives, which allowed for secondary utilization of samples (IRB No. 1104-086-359). A patient was excluded from the study if they had a diagnosis of two or more types of malignancy within the previous 5 years, withdrew consent before or during the study, or not enough samples were stored for analysis. Retrospective clinical and follow-up information was obtained from the medical records. Pre- and posttreatment samples from patients who received molecularly targeted agents were also analyzed. Blood sampling and analyses were performed after the study protocol was approved by the Institutional Review Board. All patients provided written informed consent to participate in this study. The study protocol was approved by the Institutional Review Board of Seoul National University Hospital (IRB No. 2002-070-110). All study procedures were performed in accordance with the Helsinki Declaration and its later amendments or comparable ethical standards.

### sPD-L1 ELISA

Serum was obtained by centrifugation (1300×*g* for 10 min) and then aliquoted and stored at − 80 °C until study analysis. sPD-L1 was assayed using a commercially available ELISA Kit (BMS2212, Invitrogen, Vienna, Austria) following the manufacturer’s instructions. Samples were analyzed in duplicate for each marker.

### Statistical analysis

Demographic and clinical parameters were analyzed using descriptive statistics. Differences in sPD-L1 distribution and median values in healthy donors and cancer patients were compared using nonparametric Mann–Whitney *U* tests. ROC curve analysis was used to determine the optimal sPD-L1 cutoff point for predicting treatment resistance. Relationships between treatment response and sPD-L1 or clinical variables were analyzed using *χ*^2^-tests and Student’s *t* tests. Kaplan–Meier survival analyses and Cox proportional hazards models were used to analyze PFS and OS. Analyses of correlations between sPD-L1 and circulating immune cells were performed by calculating Pearson correlation coefficients because the data met the assumptions of a normal distribution. Because the data for tissue PD-L1 expression did not follow a normal distribution, correlations with sPD-L1 were assessed using the nonparametric Spearman's rho method. Statistical analyses were performed and graphics generated using IBM SPSS statistics v.21 (IBM, Armonk, NY, USA) and Excel 2019 (Microsoft, Redmond, WA, USA) software. A p value < 0.05 was considered statistically significant.

## Supplementary Information

Supplementary Information.

## Data Availability

The datasets generated and/or analyzed in the current study are available from the corresponding author upon reasonable request.
