# Supplementary material for: Soluble PD-L1 is a predictive and prognostic biomarker in advanced cancer patients who receive immune checkpoint blockade treatment
Source: Sci Rep. 2021 Oct 5;11:19712. doi: 10.1038/s41598-021-99311-y (PMC8492653; doi:10.1038/s41598-021-99311-y)
Supplement: Supplementary file 1 — Supplementary Information. [file 41598_2021_99311_MOESM1_ESM.docx]

Research article

**Soluble PD-L1 is a predictive and prognostic biomarker in advanced cancer patients who receive immune checkpoint blockade treatment**

So Yeon Oh^1^, Soyeon Kim^2, 3^, Bhumsuk Keam^2, 4^, Tae Min Kim^2, 4^, Dong-Wan Kim^2, 4^, and Dae Seog Heo^2, 4,*^

^1^Medical Oncology, Department of Internal Medicine, Pusan National University Yangsan Hospital, Yangsan, Republic of Korea. ^2^Cancer Research Institute, Seoul National University College of Medicine, Seoul, Republic of Korea. ^3^Biomedical Research Institute, Seoul National University, Seoul, Republic of Korea. ^4^Department of Internal Medicine, Seoul National University Hospital, Seoul, Republic of Korea

Correspondence:

*Dae Seog Heo, MD, PhD

Department of Internal Medicine, Seoul National University Hospital

Address: 101, Daehak-Ro, Jongno-Gu, Seoul 03080, Republic of Korea.

Tel: +82 2 2072 2857

Fax: +82 2 762 9662

Email: heo1013@snu.ac.kr

**Supplementary data**

Supplementary Table 1. Sampling intervals according to major primary cancer types (Total N=67)

| Diagnosis | N (%) | Mean ± SD | Median (min-max) | P value* | |
| --- | --- | --- | --- | --- | --- |
| NSCLC | 17 | 18 ± 8 | 15 (14-49) | 0.005 | <0.001 |
| Melanoma | 17 | 25 ± 10 | 21 (14-49) |  |  |
| SCLC | 13 | 38 ± 9 | 42 (14-48) |  |  |
| GU cancer | 9 | 20 ± 2 | 21 (14-21) |  |  |
| Others | 11 | - | - |  |  |

*Mann-Whitney U test, significance at *p* <0.05.

Supplementary Figure 1. Comparison of mean value of sPD-L1 in healthy volunteers (n=20) and cancer patients (n=128).


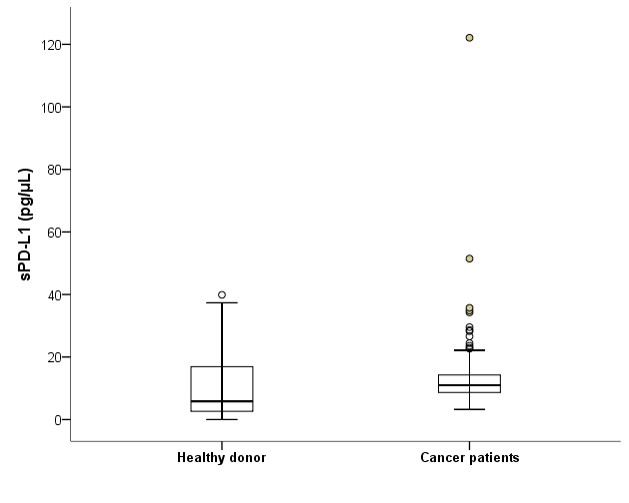


Supplementary Figure 2.

Receiver operating characteristic (ROC) curve to determine optimal cut-off level of sPD-L1 in prediction of progressive disease after immune checkpoint inhibitor treatment. In this analysis, a cut-off value of 11.0 pg/μL distinguished best between patients whose response is progressive disease from responding patients.


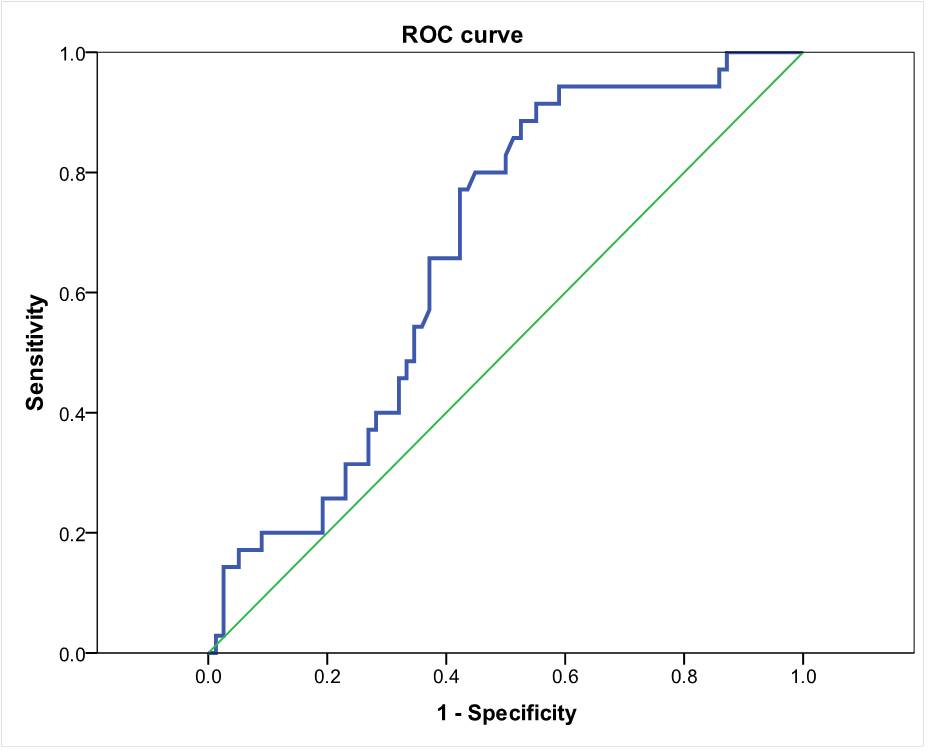


Supplementary Figure 3.

In this figure, the red bars (mean PD) are more frequently seen in the right side of the chart where cases with higher sPD-L1 level was depicted than the left side.


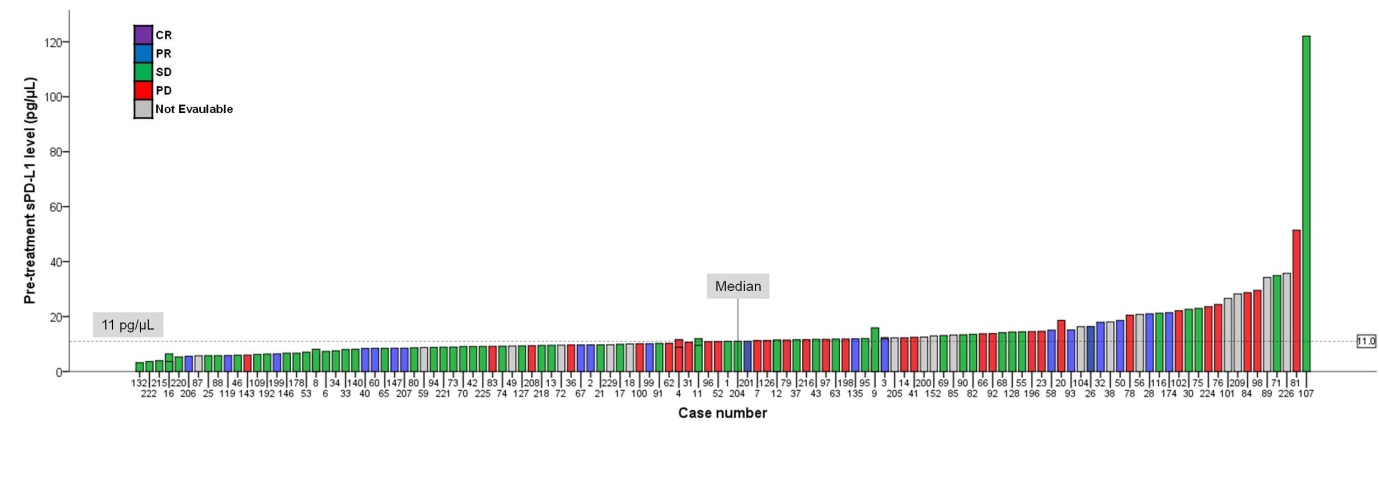


Supplementary Figure 4. Change of sPD-L1 level before treatment to after treatment according to treatment response. *CR/PR* complete response and partial response, *SD* stable disease, *PD* progressive disease.


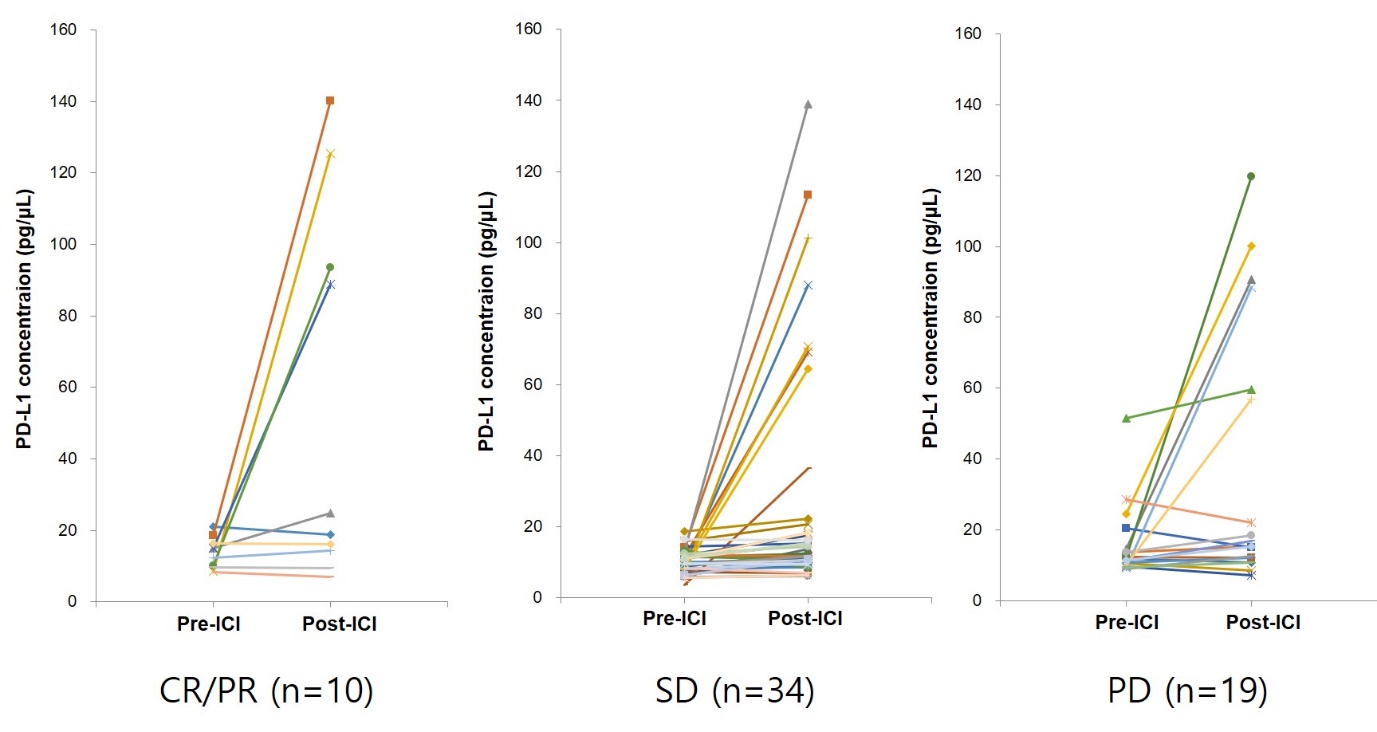


Supplementary Table 2. The change in the concentration of sPD-L1 (ΔsPD-L1) and ICI response

|  | ICI response | | Total | *P* value* |
| --- | --- | --- | --- | --- |
|  | CR, PR, SD | PD |  |  |
| ΔsPD-L1 < 0 | 11 | 6 | 17 | 0.758 |
| ΔsPD-L1 ≥ 0 | 33 | 13 | 46 |  |
| Total | 44 | 19 | 63* |  |

* Four patients’ response was not available. Significance at *p* <0.05.
